# Bedaquiline, Pretomanid, Linezolid, and Moxifloxacin: Mechanisms of Action, Drug Interactions, Adverse Effects and Use in Special Situations

**DOI:** 10.3390/microorganisms14051015

**Published:** 2026-04-30

**Authors:** Marcos Abdo Arbex

**Affiliations:** Medical School, University of Araraquara (Uniara), Araraquara 14801-320, SP, Brazil; arbexma@techs.com.br or arbexma@gmail.com

**Keywords:** tuberculosis, multidrug-resistant, BPaLM regimen, bedaquiline, pretomanid, linezolid, moxifloxacin, pharmacokinetics, drug interactions, adverse drug reactions, pregnancy, special populations

## Abstract

Tuberculosis (TB) remains a critical global public health challenge, requiring therapeutic strategies that ensure high cure rates while minimizing bacillary transmission. The 2022 World Health Organization (WHO) update for drug-resistant TB treatment prioritized a novel, 6-month, all-oral regimen composed of bedaquiline, pretomanid, linezolid, and moxifloxacin (BPaLM) as the preferred treatment for rifampicin- and multidrug-resistant tuberculosis (RR-/MDR-TB). However, the clinical success of this shortened therapy is intrinsically linked to managing complex drug–drug interactions and treatment-emergent adverse effects which may necessitate regimen modifications. This article provides a comprehensive pharmacological review of the BPaLM components, detailing their mechanisms of action, pharmacokinetics (absorption, metabolism, and excretion), and safety profiles. Furthermore, we analyze critical drug interactions—including those involving food and antacids—and provide evidence-based guidance for special clinical populations, such as pregnant and breastfeeding women, and patients with hepatic or renal impairment. Mastery of these pharmacological nuances is essential for clinicians to optimize treatment adherence and ensure improved treatment completion rates and reduced resistance emergence.

## 1. Introduction

Tuberculosis (TB) is one of the top 10 causes of death worldwide and the leading cause of death from a single infectious agent [1]. Globally, in 2024, an estimated 10.7 million people (95% uncertainty interval [UI]: 9.9–11.5 million) fell ill with TB (incident cases) and 1.23 million died from the disease (95% UI: 1.13–1.33 million). The TB incidence rate (new cases per 100,000 population per year) was 131 (95% UI: 122–141) and the case fatality rate was 11.5% [1]. Also, in 2024, a total of 164,545 people were treated for multidrug-/rifampicin-resistant TB (MDR/RR-TB). This accounted for 42% of approximately 390,000 people who developed MDR/RR-TB during that year, a rate that was almost the same as in 2023. Among the 39,000 new cases of MDR/RR-TB, 3.2% were new TB cases and 16% were previously treated [1]. The treatment success rate for drug-susceptible TB remains high, at 88%, and has improved to 71% for MDR/RR-TB. From 2000 to 2024, TB treatment is estimated to have averted 83 million deaths, despite the estimated 15,0000 (95% UI: 93,000–210,000) deaths caused by MDR/RR-TB [1].

Drug-resistant tuberculosis (DR-TB) remains a significant challenge in TB treatment and control programs worldwide [2,3]. According to the World Health Organization (WHO) classifications, drug-resistant TB (DR-TB) is defined as TB disease caused by a strain of *Mycobacterium tuberculosis* (MTB) complex that is resistant to any TB medicines; MDR/RR-TB usually refers to either multidrug-resistant (MDR-TB) or rifampicin-resistant TB (RR-TB). Multidrug-resistant tuberculosis (MDR-TB) occurs when there is resistance to isoniazid and rifampicin with or without resistance to first-line drugs; pre-extensively drug-resistant TB (Pre-XDR-TB) occurs when the strain meets the MDR/RR-TB criteria and is also resistant to any fluoroquinolone (such as levofloxacin or moxifloxacin); extensively drug-resistant TB (XDR-TB) occurs when the strain is resistant to rifampicin (and may also be resistant to isoniazid), as well as to at least one fluoroquinolone (levofloxacin or moxifloxacin) and at least one other “Group A” drug (bedaquiline or linezolid) [1,2,3].

MDR/RR-TB continues to emerge and spread due to a combination of clinical and socioeconomic factors. Primarily, suboptimal treatment practices—including the use of ineffective drug formulations, monotherapy, and premature treatment interruption—drive the development of resistance. This is further exacerbated by systemic challenges such as person-to-person transmission in overcrowded facilities (e.g., prisons and hospitals), poor medication quality, and patient-level factors, including malnutrition, diabetes, HIV, and substance use. Socioeconomic vulnerabilities like poverty and inadequate education remain critical drivers of the epidemic [1,2,4].

Furthermore, MTB has several molecular mechanisms of acquiring drug resistance, including (1) barrier mechanisms, such as cell wall permeability reduction or reverse drug transport by expression of efflux transporters (ETs); (2) degradation or inactivation enzymes; (3) modification of pathways involved in drug activation or metabolism; and (4) changes in drug targets or gene amplification [5].

Conventional MDR-TB management is associated with low treatment success rates, more severe side effects, poor adherence, prolonged treatment time, treatment interruption and considerable healthcare and societal costs. These challenges place huge pressure on individuals, communities, and national public health systems [6,7]. Moreover, MDR/RR-TB often impacts populations in low- and middle-income nations and high TB-burden countries, where healthcare infrastructure is under-resourced in managing complex drug regimens [8]. Despite the elevated rates of MDR/RR-TB incidence stabilization in recent years, the rise in XDR-TB cases poses a formidable challenge to global tuberculosis eradication initiatives [1,6,9]. In addition, studies show a huge association between MDR/RR-TB and post-TB lung disease [7].

MDR/RR-TB treatment has evolved substantially. In August 2022, data from the ZeNix trial demonstrated that 6 months of oral bedaquiline, pretomanid, and linezolid treatment produced favorable outcomes in 91% of study participants with XDR (at that time defined as resistance to rifampicin, a fluoroquinolone, and an aminoglycoside), pre-XDR, or RR-TB who did not respond to, or were unable to tolerate, standard regimens [10]; this confirmed the results of the earlier Nix-TB trial [11]. In December 2022, the TB-PRACTECAL trial showed that 6 months of oral bedaquiline, pretomanid, linezolid, and moxifloxacin (BPaLM) treatment cured 89% of people with RR-TB, irrespective of their fluoroquinolone-resistance status [12]. Based on these pivotal studies, the 2022 World Health Organization update to the drug-resistant tuberculosis treatment guidelines added and prioritized a new 6-month regimen, composed of bedaquiline, pretomanid, linezolid (600 mg) and moxifloxacin (BPaLM regimen), as a treatment of choice for MDR/RR-TB, rather than the 9-month or longer (typically 18-month) regimen [1,2,13]. The BPaLM regimen has exhibited superior therapeutic efficacy, characterized by higher success rates, enhanced tolerability, and a significant reduction in both treatment discontinuation and mortality when compared to the standard treatment regimen [14]. In addition to its clinical efficacy, the BPaLM regimen offers significant public health benefits. By utilizing a shorter, better-tolerated treatment course, it enhances patient compliance, which is critical in mitigating the development of additional resistance emerging [15].

Beyond clinical trial settings, the global implementation of the BPaLM regimen has expanded rapidly, providing essential real-world evidence on its effectiveness. According to a global analysis by Nyberg et al. (2023), by late 2022, at least 40 countries had already started implementing the 6-month BPaLM/BPaL regimen, reflecting its rapid global reach and the high demand for shorter, all-oral treatments [16].

Recent programmatic evaluations in high-burden settings have mirrored the success of controlled trials. For instance, a prospective cohort study in Sierra Leone reported a treatment success rate of 87.1% for BPaL-based regimens under routine conditions [17]. Similarly, real-world data from Thailand published in 2023 showed a success rate of 82.1% (23/28 patients), with a high rate of rapid sputum conversion within 12 weeks [18].

Furthermore, a large-scale prospective cohort study in Belarus and Uzbekistan (2025) reinforced these findings, documenting successful treatment outcomes in 95.3% (427/448) of patients with MDR/RR-TB treated with BPaL plus moxifloxacin (BPaLM) and 90.4% (207/229) of those with pre-XDR-TB treated with BPaL plus clofazimine [19]. The rapid scale-up of the BPaLM regimen underscores the critical need for Active Tuberculosis Drug-Safety Monitoring and Management (aDSM), particularly in high-burden countries. While emerging programmatic statistics are crucial, comprehensive global data are still being consolidated. In regions where full post-guideline statistics are not yet available, the WHO emphasizes that aDSM, robust pharmacovigilance, and real-time data collection remain the primary strategies to monitor long-term safety and potential resistance. Furthermore, implementing standardized pharmacovigilance frameworks is essential to bridge existing data gaps regarding long-term safety and fetal outcomes in special populations, ensuring that the high efficacy of BPaLM is matched by sustained clinical safety [20,21].

Despite this progress, there is a lack of comprehensive literature that synthesizes these emerging real-world statistics with the intricate pharmacological profiles of the BPaLM components. This review distinguishes itself by integrating recent (2023–2025) implementation data and programmatic challenges with an in-depth analysis of mechanisms of action, pharmacokinetics, and drug–drug interactions. Crucially, it provides evidence-based guidance for managing special clinical situations—such as pregnancy, breastfeeding, and hepatic or renal impairment—where data are often scarce but clinically vital for the successful global scale-up of the BPaLM regimen.

The pharmacological and clinical profiles of the BPaLM agents, including their mechanisms of action, primary adverse effects, and management strategies, are summarized in Table 1.

## 2. Linezolide (LZD)

Oxazolidinones are a class of synthetic antibacterial agents that were first produced in 1978 for their antimicrobial activity against plant pathogens. In 1987, one of these agents was developed into LZD (DuP721, PNU-100766, S-N-3-3-fluoro-4-[4-morpholinyl]phenyl-2-oxo-5-oxazolidinylmethyl-acetamide). Its chemical structure consists of an oxazolidinone ring, which is the core of its antibacterial activity, as well as functional groups such as a fluoro group on the phenyl ring, which increases lipophilicity and tissue penetration, and a morpholine group, which contributes to structural stability and interacts with bacterial protein targets. In 2000, LZD was introduced to the market for managing Gram-positive bacterial infections affecting the respiratory tract, skin, and soft tissues [22,23,24]. LZD was initially used off-label before consistent scientific evidence emerged to support its use as part of the regimen for difficult-to-treat cases of MDR- and XDR-TB [25]. Subsequently, several systematic reviews and controlled clinical trials have demonstrated the efficacy, safety and tolerability of LZD in patients with DR-TB [26,27,28,29].

According to the WHO, LZD’s clinical positioning for treating MDR-TB has undergone a dramatic transformation over the past two decades, moving from a “drug of last resort” to a foundational “Group A” medicine [30,31,32,33,34].

In its early guidelines (2006), the WHO classified LZD within Group 5 (Alternative Agents). At this stage, its efficacy against *MTB* was considered unclear; it was not recommended for routine use and was reserved only for cases where other options were exhausted [30]. In 2008, LZD was formally introduced into the WHO guidelines for MDR-TB treatment. Since its introduction, its classification and clinical role have evolved significantly [31]. By 2011, LZD had been initially classified as a Group 5 drug, a category reserved for anti-TB agents with limited clinical data regarding long-term safety and efficacy. At this stage, it was recommended only as a drug of last resort when an effective regimen could not otherwise be constructed [32]. In 2016, following an expanded evidence base, the WHO reallocated LZD to Group C (“Other core second-line agents”). This represented a significant shift, as it prioritized LZD over several traditional second-line injectable agents, provided that clinical monitoring for adverse events (such as neuropathy and myelosuppression) was available [33]. A landmark Rapid Communication published in 2018 redefined the MDR-TB treatment hierarchy. LZD was upgraded to Group A (“Medicines to be prioritized”), alongside bedaquiline and fluoroquinolones. It became a mandatory component of the “longer” all-oral regimens unless contraindicated [34]. In 2022, the WHO consolidated guidelines now recommend a 6-month BPaLM regimen as the preferred treatment for most MDR-TB patients, further cementing linezolid’s role in modern, all-oral therapy [13].

Despite exhibiting bacteriostatic properties in vitro against MTB—including MDR and XDR strains—LZD demonstrates robust efficacy in both in vitro and in vivo models. LZD demonstrates excellent penetration into target tissues such as the lungs and alveolar macrophages, while achieving therapeutic, albeit variable, concentrations in the central nervous system [35,36,37]. In clinical cases of cavitary pulmonary TB, LZD penetrates tuberculosis lesions and infected alveolar macrophages. The results suggest that while LZD is traditionally classified as bacteriostatic, it exhibits potent concentration-dependent activity against both actively replicating and slowly growing (semi-dormant) bacilli. This contribute to the **sterilization** of tuberculosis lesions and the prevention of relapse, which are critical factors for treatment success and curtailing disease transmission [35,36,37].

LZD stands as one of the cornerstones in managing MDR-TB and XDR-TB, offering distinct pharmacological advantages over alternative therapies. A primary benefit is its near-complete oral bioavailability, which ensures therapeutic equivalence between oral and parenteral administration. This flexibility facilitates the transition to outpatient care, significantly reducing hospital dependency during prolonged treatment. Furthermore, its superior pulmonary distribution allows for optimal drug concentrations directly at the infection site, surpassing the efficacy of agents with more restricted tissue penetration [38]. A further benefit lies in its unique mechanism of action, which minimizes the possibility of cross-resistance—a common issue with other anti-TB drugs like fluoroquinolones and aminoglycosides. Additionally, bedaquiline, delamanid, and moxifloxacin carry risks of cardiotoxicity through QTc interval prolongation, whereas LZD lacks this specific effect. While this safety profile is advantageous for patients with pre-existing cardiac conditions, LZD is a complementary component rather than a direct substitute for these agents. Maintaining an effective regimen requires a careful balance of drugs from different classes to ensure both efficacy and safety [35,36,37].

The optimization of LZD dosing has been a focal point of recent research to balance its potent efficacy with its dose- and duration-dependent toxicity. Data from the landmark Nix-TB and ZeNix trials have redefined the clinical approach to LZD use within the BPaL-based regimens. While the initial Nix-TB study employed a dose of 1200 mg daily for 26 weeks, it was associated with high rates of peripheral neuropathy (81%) and myelosuppression (48%) [11]. Subsequent results from the ZeNix trial demonstrated that reducing the LZD dose to 600 mg daily for 6 months—or even 600 mg for a shorter duration of 9 weeks—maintained high treatment success rates (above 84–90%) while significantly reducing the incidence of adverse events [10]. Consequently, current evidence supports starting LZD at 600 mg daily as part of the BPaLM regimen, with the flexibility for dose reduction (to 300 mg) or interruption if toxicities arise, without compromising the overall sterilization of tuberculosis lesions [3,10,11,39].

LZD exhibits potent bacteriostatic activity against MTB, with a minimum inhibitory concentration (MIC) typically ranging from 0.125 to 1.0 µg/mL for both drug-susceptible and -resistant strains [27,28].

### 2.1. LZD Mechanism of Action

LZD exerts its antibacterial activity through a unique mechanism of protein synthesis inhibition. Unlike most other protein synthesis inhibitors (such as aminoglycosides or macrolides) that interfere with peptide chain elongation or cause mRNA misreading, LZD acts at the earliest stage of the translation process [4,40,41,42].

The drug binds to the 23S ribosomal RNA (rRNA) of the 50S subunit within the peptidyl transferase center (PTC). Specifically, it targets the V domain of the 23S rRNA, preventing the formation of the 70S initiation complex. This complex is essential for the 30S and 50S subunits’ association with messenger RNA (mRNA) and fMet-tRNA. By obstructing this assembly, LZD effectively halts the initiation of bacterial protein synthesis [41,43]. However, the impact of linezolid on ribosomal RNA is not limited to bacteria; it also inhibits mitochondrial protein synthesis in human cells, which can lead to adverse effects associated with mitochondrial dysfunction. These include and result in lactic acidosis, myelosuppression, and peripheral and optical neuropathy [38,44].

### 2.2. LZD Mechanisms of Resistance

Specific risk factors facilitate the development of LZD resistance. Notably, dose reductions—if necessitated by mitochondrial toxicity—result in suboptimal drug exposure. This inadequate concentration fails to suppress bacterial replication, thereby providing a selective advantage for the emergence of resistant mutants. Furthermore, the clinical practice of adding linezolid to failing treatment regimens significantly exacerbates the risk of selecting for drug-resistant strains [45]. In *MTB*, LZD resistance is predominantly driven by target-site modifications that disrupt the drug’s binding to the 50S ribosomal subunit. The primary mechanism involves spontaneous point mutations in the 23S rRNA gene, specifically at the PTC. Mutations such as G2061T and G2576T (H37Rv numbering) are clinically significant as they alter the ribosomal architecture, drastically reducing the binding affinity of the oxazolidinone ring [45,46,47].

### 2.3. LZD Pharmacokinetics

LZD exhibits exceptional pharmacokinetics, characterized by 100% oral bioavailability and extensive tissue distribution to sites, including the cerebrospinal fluid, alveolar macrophages, and bone [40,48,49]. Its absorption is not significantly affected by food or the co-administration of antacids, such as magnesium or aluminum hydroxide; clinically stable patients can therefore seamlessly transition from intravenous (IV) to oral (PO) administration [34,40,42,43,48,49]. Following a 600 mg dose, peak serum concentrations (Cmax) of 15–27 mg/L are typically achieved within 0.5 to 2 h. Although high-fat meals may slightly delay the time to maximum concentration (Tmax) and marginally reduce Cmax, the total area under the curve (AUC) remains unaffected [3,48,49,50].

The plasma protein-binding of LZD is approximately 31%, and it primarily involves albumin. Its volume of distribution (Vd) aligns with total body water (40–50 L), while the plasma half-life ranges from 5.0 to 7.0 h [3,42,43,44,48,49,50]. LZD is primarily metabolized in the liver via non-enzymatic oxidation, independent of the cytochrome P450 (CYP450) enzyme system, thus avoiding CYP450 isoform inhibition. The drug is converted into two primary inactive metabolites: hydroxyethyl glycine and aminoethoxyacetic acid. Renal excretion is the major elimination pathway, with 30% of the dose excreted as the parent drug, 40% as hydroxyethyl glycine, and 10% as aminoethoxyacetic acid. Conversely, fecal excretion is minimal. Total LZD clearance is estimated to be between 100 and 200 mL/min. Although its metabolites are primarily eliminated renally, non-renal pathways account for approximately 65% of the total clearance of the parent drug. Mean renal clearance of the parent drug is roughly 40 mL/min, a rate suggestive of net tubular reabsorption; notably, significant inter-individual variability in clearance has been observed, particularly regarding the non-renal component [38,48,49,50,51].

### 2.4. LZD Adverse Effects (AEs)

Managing AEs in TB therapy is challenging. Most AEs are minor and can be managed without treatment discontinuation, but some can be major or life-threatening, leading to either modification or discontinuation of the treatment regimen and death if not recognized and treated promptly. Regarding LZD, its long-term use can be limited by dose-dependent toxicities, as detailed in the previous section. The clinical challenge lies in managing these interruptions through the dosing optimizations and proactive reduction strategies already discussed, which are essential to mitigate severe adverse effects while maintaining therapeutic efficacy [52,53,54].

According to the WHO, the most common AEs associated with LZD include gastrointestinal disturbances, myelosuppression, optic neuropathy, and peripheral neuropathy [3]. In the short term, the most frequently reported reactions are diarrhea, nausea, vomiting, headache, and transient elevations in liver enzymes [3,43]. These symptoms typically require only supportive care; notably, hepatic enzyme elevations are rarely severe enough to warrant drug discontinuation [3,49,55].

LZD-induced myelosuppression—encompassing anemia, thrombocytopenia, leukopenia, and pancytopenia—is primarily attributed to the reversible, dose-dependent inhibition of mitochondrial protein synthesis [45,49]. While these hematologic abnormalities typically manifest after at least two weeks of therapy, the risk of myelotoxicity appears significantly lower in patients receiving daily doses of ≤600 mg, even during prolonged treatments exceeding 20 months [55]. Recovery of bone marrow function is generally observed within one to three weeks post-discontinuation. Consequently, weekly hematologic monitoring is essential for patients on LZD-containing regimens [3,45,49]. Beyond monitoring, clinical strategies to mitigate toxicity include the use of erythropoietin or a dose reduction to 300 mg daily, which has proven effective in managing anemia without compromising therapeutic efficacy [55,56,57].

Neurologic toxicity associated with linezolid primarily manifests as peripheral and optic neuropathy [58,59]. These adverse effects are both time- and dose-dependent, typically emerging after a median duration of 5–11 months [58,59]. Optic neuropathy is characterized by blurred vision, ocular pain, visual impairment, scotomas, dyschromatopsia (color-perception impairment), and photophobia [58,59]. The underlying mechanism is attributed to mitochondrial dysfunction, where LZD inhibits mitochondrial protein synthesis and disrupts cellular energy production [44,60]. Following LZD discontinuation, visual recovery generally occurs within 1 to 3 months [58,59]. Consequently, routine ophthalmologic screening is recommended for patients undergoing prolonged therapeutic courses [3,58,59]. LZD-associated peripheral neuropathy is generally irreversible and exhibits a strong correlation with treatment duration and cumulative dosage. Clinical manifestations typically follow a ‘glove-and-stocking’ distribution, characterized by numbness, paresthesia, tingling, and impaired perception of pain, temperature, and light touch, alongside loss of proprioception [58,59]. Although the exact pathogenesis remains elusive, current theories suggest that mitochondrial toxicity affects motor and sensory nerves, leads to myelin sheath degradation, or inhibits Schwann cell proliferation [38,52,53,54]. The high incidence observed in TB patients may stem from prolonged therapy or the synergistic neurotoxic effects of concomitant anti-TB agents [39,52,53]. Symptomatic management typically involves the use of amitriptyline, gabapentin, or pyridoxine [58,59].

In addition, according to the WHO, LZD was also reported with occasional reactions like pseudomembranous colitis, vaginal candidiasis, hypoglycemia, serotonin syndrome and lactic acidosis; there have also been reports of arrhythmia (tachycardia), transient ischemic attacks, pancreatitis, seizures and uncommon reactions like Stevens–Johnson syndrome, angioedema and alopecia [3].

Serotonin syndrome (SS) is a rare but potentially fatal condition resulting from overstimulation of central and peripheral 5-HT receptors due to serotonergic drug exposure. This syndrome often follows the initiation of a new agent or a dosage escalation. Although LZD is a weak, reversible, and non-selective monoamine oxidase inhibitor, its co-administration with selective serotonin reuptake inhibitors (SSRIs) can precipitate SS. Diagnosis remains clinical and challenging, as no definitive confirmatory tests exist. The clinical triad includes neuromuscular hyperactivity (e.g., tremors, hyperreflexia, and myoclonus), autonomic instability (hyperpyrexia), and altered mental status. In LZD-related cases, symptoms typically emerge within the first week of therapy. A pre-emptive dose reduction or discontinuation of serotonergic agents should therefore be considered during linezolid treatment [49,59,61].

LZD-induced lactic acidosis is an infrequent yet severe metabolic complication, predominantly observed in adult patients undergoing prolonged therapy for MDR-TB, typically exceeding 28 days. This condition is characterized by a significant decrease in systemic pH and an accumulation of L-lactate. The pathogenesis is rooted in mitochondrial toxicity, where linezolid interferes with mitochondrial protein synthesis. Specifically, this inhibition leads to a reduction in cytochrome c oxidase activity—an enzyme partially synthesized by mitochondrial ribosomes. Clinical manifestations include recurrent nausea, vomiting, and muscle weakness. Because these symptoms can be mistaken for other anti-TB drug toxicities, a high index of suspicion is required. Laboratory findings typically reveal elevated anion gap metabolic acidosis with significantly increased serum lactate levels. If left unmanaged, this toxicity can escalate to multi-organ failure, involving hepatic and renal dysfunction, and may ultimately be fatal. Management necessitates the immediate LZD discontinuation, which usually leads to acidosis resolution within 2 weeks; however, severe cases may require supportive care such as hemodialysis or bicarbonate infusion [44,49,58,62,63].

### 2.5. LZD Interactions

LZD presents significant potential for drug–drug interactions. Although it does not involve the cytochrome P450 (CYP450) enzyme system, it acts as a reversible, non-selective monoamine oxidase inhibitor. Consequently, LZD’s co-administration with other drugs may increase the risk of severe clinical conditions and linezolid-induced toxicities with serotonergic or adrenergic agents may precipitate severe clinical toxicities [3,42]. Combinations with other drugs may increase the risk of severe clinical conditions and linezolid-induced toxicities [3,48].

**Risk of Hypertensive Crisis:** LZD should not be co-administered with vasopressor agents unless essential. Direct or indirect-acting sympathomimetics (e.g., pseudoephedrine, epinephrine, and norepinephrine), adrenergic bronchodilators, and dopaminergic agents (e.g., dopamine and dobutamine) require careful dose titration to prevent acute hypertensive episodes. Similarly, caution is advised when using pethidine or buspirone [64,65,66].

**Increased risk of serotonin syndrome**: Because linezolid is a weak, reversible MAO inhibitor, there is a theoretical increased risk when co-administered with SSRIs, Tricyclic Antidepressants (TCAs), serotonin 5-HT1 receptor agonists, bupropion, anti-seizure medication, opioid analgesics, buspirone, antiemetics, anti-Parkinson’s medication, sympathomimetic agents, vasopressive agents, dopaminergic agents and common medications used for influenza or congestion and bought over the counter, such as dextromethorphan, pseudoephedrine, diphenhydramine or guaifenesin. However, clinical reports of serotonin syndrome in patients receiving linezolid remain uncommon; nevertheless, healthcare providers should maintain active vigilance and monitor for symptoms when these agents are used concomitantly [3,58,64,65,66].

**Hematologic and Metabolic Synergies:** The risk of pancytopenia is exacerbated when LZD is associated with other myelosuppressive agents, such as zidovudine or co-trimoxazole. Furthermore, LZD may increase the risk of lactic acidosis—a known mitochondrial toxicity—when combined with metformin or nucleoside reverse transcriptase inhibitors (NRTIs) like lamivudine, zidovudine, and abacavir [3,64,66].

**Dietary Tyramine Interaction:** Due to LZD’s role as a monoamine oxidase inhibitor, patients should avoid foods with high tyramine content to prevent hypertensive responses. Proscribed items include aged cheeses, fava beans, cured or dried meats, pickled foods (e.g., sauerkraut and kimchi), soy-based sauces (e.g., teriyaki and fish sauce), red wine, and tap beers [3,64,66].

**Monoamine oxidase inhibitors:** Although LZD acts as a reversible and non-selective monoamine oxidase inhibitor, its antibacterial dosages are insufficient to exert antidepressant effects. However, its inhibitory profile poses significant safety concerns regarding pharmacodynamic interactions. Due to a paucity of clinical data on concurrent monoamine oxidase inhibition, LZD is contraindicated with specific agents such as isocarboxazid, moclobemide, phenelzine, and selegiline. In cases where co-administration with other drugs potentially affected by monoamine oxidase inhibition is unavoidable, rigorous clinical surveillance and continuous blood pressure monitoring are mandatory [3,48,64,66].

**Rifampicin:** Concomitant administration with rifampicin, a potent inducer of metabolic pathways, significantly alters LZD pharmacokinetics. Studies have shown that rifampicin decreases the mean LZD Cmax by 21% [90% CI: 15–27%] and the mean AUC by 32% [90% CI: 27–37%]. Evidence suggests this interaction is driven by the induction of P-glycoprotein (P-gp) and potentially other non-CYP-mediated pathways, which accelerate LZD clearance. This reduction in LZD concentration may potentially compromise therapeutic efficacy in TB treatment [40,49,59,66].

**Warfarin:** When warfarin is administered at steady-state during LZD therapy, a 10% reduction in the mean maximum International Normalized Ratio (INR) and a 5% reduction in the AUC of the INR have been observed [60]. Current clinical data are insufficient to fully assess the significance of these findings; however, close monitoring of coagulation parameters is advised when initiating or discontinuing LZD in patients on anticoagulation therapy [66,67].

### 2.6. LZD Specific Patient Population

**Breastfeeding:** LZD is excreted into human breast milk, with clinical data suggesting that infant exposure through breastfeeding reaches approximately 6% to 9% of the standard pediatric dose. While specific studies on the long-term effects in breastfed infants are lacking, data indicate that linezolid levels in breast milk are trivial, leading to minimal systemic exposure in the infant. Although diarrhoea and vomiting have been identified as the most frequent adverse reactions in infants treated directly with LZD, many experts consider the drug’s use to be probably safe during breastfeeding. Consequently, the decision to continue breastfeeding during maternal tuberculosis therapy requires a careful risk–benefit analysis, weighing the immunological and nutritional advantages of breast milk against the potential for infant drug toxicity [3,64,66,68].

**Pregnancy:** Clinical data regarding LZD use in pregnancy remain limited; however, current evidence has not established a definitive link to congenital malformations or direct fetal harm. Nonetheless, reproductive toxicity has been observed in animal models, necessitating caution. The physiological changes in pregnancy—specifically hemodilution, which lowers baseline hemoglobin, and an increased predisposition to peripheral neuropathy—may exacerbate LZD’s known AE profile. Consequently, LZD should be reserved for pregnant patients only when the therapeutic benefits for the mother clearly outweigh the potential risks to the fetus, following a rigorous risk–benefit assessment [3,64,66,69].

**Hepatic impairment**: LZD pharmacokinetics remain unchanged in mild-to-moderate hepatic impairment (Child–Pugh class A or B), and no dosage adjustment is recommended. The pharmacokinetics of LZD in patients with severe hepatic insufficiency (i.e., Child–Pugh class C) have not been evaluated. As LZD is metabolized by a non-enzymatic process, hepatic function impairment is not expected to significantly alter its metabolism. However, the risk of hematological toxicity increases in patients with cirrhosis [3,64,66,70].

**Renal impairment:** LZD pharmacokinetics of the parent drug are relatively preserved in mild-to-moderate renal impairment. However, in patients with severe renal insufficiency (CrCl < 30 mL/min), there is a significant 7–8-fold increase in the plasma concentrations of the two primary metabolites. This accumulation is clinically relevant as it may increase the risk of adverse effects, necessitating careful monitoring in this population [64,66].

**Fertility**: In animal studies, LZD reduced male fertility. These effects were reversible in adult animals but did not reverse in juvenile animals treated with LZD for nearly the entire period of sexual maturation. There were no adverse effects on female fertility. The effect on fertility in humans is unknown. The potential risk of reduced male fertility should be taken into account when treating adolescents and post-pubertal boys [66].

## 3. Moxifloxacin (MFX)

Discovered in the early 1960s by George Lesher, nalidixic acid—a 1,8-naphthyridine derivative—marked the inception of the quinolones, which have since become one of the most critical classes of antimicrobial agents. Its structure was derived from a byproduct (7-chloro-1-ethyl-1,4-dihydro-4-oxoquinoline-3-carboxylic acid) synthesized during the development of the antimalarial chloroquine. Although initially utilized for Gram-negative urinary tract infections, nalidixic acid exhibited poor tissue penetration and suboptimal efficacy against systemic infections. The subsequent development of fluoroquinolones (FQs) in the 1980s, achieved by incorporating a fluorine atom into the quinolone core, represented a significant therapeutic breakthrough.

This evolution led to several generations of antimicrobial agents: ciprofloxacin (CFX) and ofloxacin (OFX) (second generation); levofloxacin (LFX) (third generation); and moxifloxacin (MFX) and gatifloxacin (GFX) (fourth generation). These broad-spectrum antibiotics demonstrate potent activity against diverse pathogens, including *Mycoplasma*, *Chlamydia*, and *Mycobacterium* species. Due to their ability to traverse bacterial cell membranes—primarily via porins—FQs are particularly effective against intracellular pathogens. Within this class, moxifloxacin offers an expanded spectrum against Gram-positive bacteria and atypical pathogens, playing a pivotal role in MDR-TB management [5,71,72,73,74].

In 2000, a landmark meeting in Cape Town involving researchers, pharmaceutical companies, and funding agencies culminated in the Cape Town Declaration. This initiative established a strategic commitment to license at least one novel anti-tuberculosis agent by 2010. The subsequent establishment of the Global Alliance for TB Drug Development significantly revitalized the field, fostering a robust pipeline of promising candidates. Among these, MFX, a fourth-generation fluoroquinolone (FQ), emerged as a pivotal agent capable of fulfilling the Declaration’s ambitious goals.

While early FQs lacked the requisite bactericidal and sterilizing activity to significantly shorten TB treatment, MFX possesses distinct structural modifications compared to OFX and CFX. These enhancements significantly contribute to the sterilizing activity of combination regimens, increasing the likelihood of achieving a relapse-free cure and supporting shorter treatment durations. Although within-class cross-resistance, it has been argued that the superior bactericidal activity and lower MICs of MFX allow it to remain effective against MTB and DR-TB strains where CFX and OFX may fail. Notably, MFX stands out for its exceptional potency, comparable to that bactericidal activity and sterilizing efficacy of isoniazid [75,76,77,78].

Structurally, MFX is distinguished from OFX and by the incorporation of a methoxy group at the C-8 position and a diazabicyclononyl ring at the C-7 position of the quinolone nucleus. The C-8 methoxy substitution is particularly critical, as it broadens the antibacterial spectrum to include Gram-positive pathogens and significantly enhances activity against mycobacteria; it has a lower propensity for MTB to develop resistance against the drug, as well as for XDR-TB patients, provided it exhibits a MIC of <2 mg/L. Furthermore, MFX exhibits a favorable pharmacokinetic profile, characterized by a large volume of distribution and superior penetration into the cerebrospinal fluid (CSF), bone, alveolar macrophages, and epithelial lining fluid. These pharmacokinetic properties make it a crucial component in therapeutic regimens for XDR-TB patients. This profile confers exceptional bactericidal and sterilizing activity, even against ofloxacin-resistant strains. The potent in vitro activity of moxifloxacin against MTB, validated in both murine models and clinical monotherapy studies, suggests its potential as a cornerstone for shortened treatment regimens. Experimental data in mice further demonstrate that moxifloxacin-containing combinations achieve higher bactericidal rates than standard therapy, enabling cure without relapse in shorter durations [29]. MFX is therefore one among a small number of highly bactericidal anti-TB drugs and it contributes to the development of more effective treatment regimens. This is due to its excellent in vitro and in vivo bactericidal activity, even rivaling the potency of isoniazid and demonstrating the same sterilizing activity [76,78,79,80,81,82].

In 1996, the WHO designated FQs, such as ciprofloxacin (CFX) and OFX, second-line antitubercular agents, primarily for patients with resistance or intolerance to standard first-line therapies [83]. By 2003, FQs were established as a foundational ‘backbone’ component of MDR-TB therapy [84].

The classification of these agents evolved significantly in subsequent years. In 2008, MFX, LFX and OFX were categorized within Group 3 for MDR-TB regimens [25]. By 2011, later-generation FQs were recognized as critical components of MDR-TB treatment due to their association with improved clinical outcomes. Furthermore, WHO guidelines highlighted the superior bactericidal activity of later-generation FQs, such as LFX, MFX, and GTX, over earlier agents like OFX and CFX [85].

A pivotal shift in MDR-TB treatment prioritization occurred in 2016, when the rankings of FQs and second-line injectable drugs were re-evaluated. FQs were established as the primary core of treatment regimens due to their dual bactericidal and sterilizing properties, alongside a superior safety profile compared to injectables, which possess only bactericidal activity and higher toxicity. Consequently, FQs were reclassified into Group A, while injectables were moved to Group B [33]. Additionally, the WHO recommended phasing out OFX and explicitly stated that CFX should no longer be used due to the limited evidence of its effectiveness [33]. Most recently, in 2022 MFX was integrated into the 6-month BPaLM regimen, cementing its role as a cornerstone of modern, short-course therapy [13]. Although MFX is preferred in this regimen due to its potent bactericidal activity and lower MICs, LFX remains the primary alternative. While both agents share similar mechanisms, MFX has demonstrated superior sterilizing activity in certain experimental models. However, LFX should be considered in place of MFX when there is a high risk of cardiotoxicity, as it carries a lower potential for QTc interval prolongation. Furthermore, according to WHO guidelines, LFX is the recommended substitute if MFX is not tolerated or if specific resistance patterns suggest a benefit in switching, thereby maintaining the regimen’s efficacy while prioritizing patient safety [3,73,74,77,81,82].

### 3.1. MFX Mechanism of Action

MFX exerts its bactericidal activity by targeting DNA gyrase (a Type II topoisomerase), an essential enzyme for *MTB* survival and replication. DNA gyrase is responsible for introducing negative supercoils into the bacterial DNA molecule, a process vital for maintaining DNA topology during transcription, replication, and recombination [76,77,86,87].

In most bacteria, FQs target both DNA gyrase and topoisomerase IV. However, in the *MTB*, topoisomerase IV is notably absent, making DNA gyrase the sole target for this drug class [70,71,76]. MFX binds to the enzyme–DNA complex, stabilizing the cleavage complexes and preventing the re-ligation of the DNA strands. This stabilization results in the formation of persistent double-strand breaks, which lead to the collapse of replication forks. These events trigger irreversible chromosomal fragmentation and the subsequent loss of cellular integrity, serving as the primary drivers of rapid bacterial cell death [76,77,86,87,88].

The superior efficacy of MFX compared to earlier quinolones, such as CFX, is attributed to its higher binding affinity for mycobacterial DNA gyrase and its unique ability to eradicate bacilli in both replicating and dormant states [77,78,82]. This sterilizing activity is crucial for the clinical management of tuberculosis, as it targets the persistent sub-populations of bacteria responsible for treatment failure and disease relapse [82,89]. Furthermore, MFX demonstrates potent bactericidal activity that is less dependent on active protein synthesis than other classes. This property contributes to its role as a cornerstone of modern MDR-TB therapy, showing favourable activity comparable to later-generation fluoroquinolones in targeting actively replicating and semi-dormant bacilli [76,77,81].

### 3.2. Mechanisms of Resistance

Bacterial resistance occurs rapidly when an MFX is used as monotherapy or when it is included in regimens that have failed. The main mechanism of MFX resistance is mutation in DNA gyrase (gyrA and gyrB), especially in gyrA. Mutations that occur in the *gyrA* gene codons 94, 91 and 90 will cause a higher risk of resistance. Additionally, double mutation in the gyrA or concomitant gyrA and gyrB mutations have been reported. In addition, excessive expression of the pstB transporter protein can accelerate resistance [5,76,77,78,86,90].

### 3.3. MFX Pharmacokinetics

MXF is administered via oral tablets or intravenous (IV) solutions, exhibiting high oral bioavailability (~90%) and a plasma protein binding rate of 40%. Gastrointestinal absorption after the WHO-recommended oral dose of 400 mg is efficient and unaffected by food intake. Following oral administration, a Cmax of approximately 4.5 ± 0.5 mcg/mL is achieved at a median of 3 h. The AUC, which represents the total systemic drug exposure, exhibits a linear and dose-proportional relationship within the therapeutic range. After a single 400 mg dose, this is approximately 35–48 mg·h/L. Both Cmax and AUC values are comparable between oral and IV routes, reflecting linear pharmacokinetics where plasma levels increase proportionally with dose. This pharmacokinetic profile ensures a seamless transition between administration routes. For patients with dysphagia, tablets remain effective when crushed and suspended in water for immediate use. MFX exhibits extensive tissue distribution, achieving concentrations in the bronchial mucosa, lung tissue, alveolar macrophages, and epithelial lining fluid that surpass the MIC values for activity against MTB and for the WHO-defined critical concentration of 2.0 mg/L. Regarding central nervous system penetration, peak CSF concentrations and AUC reach approximately 50% and 75% of plasma values, respectively. Its pharmacokinetic profile, characterized by an elimination half-life of 11.5 to 15.3 h, facilitates a convenient once-daily dosing regimen. In addition, evidence suggests that MFX may retain clinical utility in OFX-resistant tuberculosis, provided the MIC remains below the 2.0 mg/L threshold. MFX does not undergo Phase I metabolism via the cytochrome P450 (CYP450) system, nor does it act as an inducer or inhibitor of these enzymes. Metabolism occurs primarily through Phase II pathways, with approximately 50% of the dose undergoing hepatic sulfate and glucuronide conjugation. The remaining 45% is excreted unchanged in both urine and feces. Specifically, glucuronide conjugates are eliminated renally, while sulfate conjugates are recovered in the feces. Furthermore, MFX pharmacokinetics are highly consistent across diverse demographic groups, showing no clinically significant variations related to age or gender [3,35,78,80,82,87,91,92,93,94].

### 3.4. MFX Adverse Effects

MFX is generally well-tolerated in patients with MDR-TB, with a safety profile comparable to other FQs. However, prolonged treatment—often necessary in MDR-TB regimens—requires careful monitoring due to the risk of cumulative or late-onset toxicities. Adverse effects following the use of MFX may occur within hours to weeks after a systemic drug is initiated; they have occurred in all age groups and in patients without pre-existing risk factors for such adverse reactions. At the first signs of any serious reactions, it is recommended that the drug be immediately discontinued [87,95,96].

**Gastrointestinal effects**: The most common side effects of MFX are gastrointestinal. Patients can present with nausea, vomiting, aerophagy, anorexia, abdominal discomfort, and diarrhea. Regarding its safety profile, moxifloxacin-induced hepatotoxicity has been documented, ranging from asymptomatic elevations in serum transaminases to rare, idiosyncratic occurrences of fulminant hepatitis. Clinical vigilance is recommended, particularly in patients with pre-existing hepatic impairment. Additionally, although infrequent, cases of Clostridioides difficile-associated diarrhea (pseudomembranous colitis) have been reported [3,25,76,82,86,94,97].

**Central nervous system (CNS) effects**: Neurological side effects include dizziness, headache, insomnia, tremors, and mood disorders in patients treated with FQs, including MFX. Hallucinations, delusions, and convulsions are rare. Greater attention should be paid to these effects in elderly patients and in those using theophylline or non-steroidal anti-inflammatory drugs (NSAIDS). Compared to other fluoroquinolones, moxifloxacin (along with gatifloxacin) exhibits a higher binding affinity for GABA receptors than levofloxacin or ciprofloxacin, which may theoretically translate into a higher risk of neurological symptoms. However, in clinical practice, the incidence of severe CNS events remains low across the class, and the choice between agents often depends more on their cardiovascular profile (QT prolongation) and antimicrobial spectrum than on differences in neurotoxicity.

In patients with a history of CNS disorders, caution is warranted [3,25,76,86,97,98,99,100,101].

**Skin reactions and allergies**: Some patients treated with FQs may experience erythema, pruritus, and skin rash. Phototoxicity can occur when patients are exposed to ultraviolet light. Urticaria, angioedema, anaphylactic reactions, and vasculitis are uncommon but possible [25,88,94,98,100].

**Musculoskeletal effects**: Common musculoskeletal complaints during prolonged MFX therapy include arthralgia, myalgia, and muscle spasms. Additionally, the drug should be used with extreme caution in patients with myasthenia gravis, as it may exacerbate muscle weakness and potentially lead to life-threatening respiratory failure. Like other FQs, MFX is associated with an increased risk of tendinitis and tendon rupture, most commonly affecting the Achilles tendon. This risk is particularly elevated in patients over 60 years of age, those with renal impairment, those with rheumatoid arthritis or individuals receiving concomitant corticosteroid or hemodialysis therapy. Clinical studies have shown that tendon damage can occur as early as 48 h after initiating treatment or be delayed for several months after discontinuation. Due to the potential for arthropathy observed in weight-bearing joints in juvenile animal models, the use of moxifloxacin in children and adolescents is generally restricted unless the benefits clearly outweigh the risks, as in cases of MDR-TB. Clinical guidelines recommend immediate discontinuation of the drug at the first sign of tendon pain or inflammation [3,97,98,99,100,102,103].

**Cardiovascular effects:** The most significant cardiac concern is the prolongation of the QTc interval, which can lead to life-threatening arrhythmias like Torsades de Pointes. This risk is heightened when MFX is co-administered with other QT-prolonging drugs common in MDR-TB therapy, such as clofazimine or bedaquiline. This is a rare event. Electrocardiographic QT interval prolongation is dose-dependent. Patients with kidney failure, liver failure, cardiomyopathy, hypomagnesemia, or hypokalemia, as well as those using class IA (procainamide and quinidine) or class III antiarrhythmic drugs (amiodarone and sotalol), together with those using terfenadine, erythromycin, cisapride, or tricyclic antidepressants, should receive special attention. Regular electrocardiogram (ECG) monitoring is recommended at baseline and periodically during treatment [3,74,97,98,99,100,104].

**Endocrine effects:** Changes in glycemia levels, including symptomatic hypoglycemia and, less commonly, hyperglycemia, have been reported in patients with diabetes who use FQs in conjunction with oral hypoglycemic agents or insulin. While the precise pathophysiology of FQ-induced dysglycemia remains to be fully elucidated, it is hypothesized to involve a sulfonylurea-like interaction with ATP-sensitive potassium channels in pancreatic beta cells. This interaction facilitates calcium influx, subsequently triggering insulin secretion. Furthermore, FQs may exacerbate this effect by inhibiting cytochrome P450 isoenzymes, thereby impairing the metabolic clearance of several concomitant antidiabetic medications [97,98,105].

**Urinary tract effects**: Interstitial nephritis, characterized by the presence of eosinophils and crystals in urine, can occur in patients treated with FQs, including MFX. These are rare events. The risk of crystalline nephropathy is significantly exacerbated by high-dose regimens or inadequate dosage adjustments in patients with impaired renal function. Contributing factors include insufficient hydration, advanced age, and the concomitant administration of nephrotoxic agents, such as NSAIDs, renin-angiotensin system inhibitors, and diuretics [86,98].

**Uncommon adverse effects:** These include peripheral neuropathy, insomnia, disturbances in mental abilities, retinal detachment, aortic aneurysm rupture and aortic dissection in patients with Marfan syndrome [3,97,98].

### 3.5. MFX Interactions

MFX undergoes minimal hepatic metabolism, primarily through Phase II glucuronide and sulfate conjugation, rather than the CYP450 enzymatic system. This metabolic pathway is distinct from other antimicrobials, as moxifloxacin does not significantly inhibit or induce CYP450 isoenzymes, thereby minimizing the risk of clinically significant drug–drug interactions associated with this system [94,106,107].

**Dietary and Antacid Interactions:** Unlike several other FQs, the gastrointestinal absorption of MFX is not significantly impaired by food intake [108]. However, the concomitant administration of antacids, sucralfate, or multivitamins containing divalent or trivalent cations (e.g., aluminum, iron, zinc, or magnesium) markedly reduces the drug’s absorption and systemic concentration [94]. Consequently, moxifloxacin should be administered at least 4 h before or 8 h after these products [91,109]. Notably, H2-receptor antagonists do not interfere with its absorption. Furthermore, unlike other quinolones, the extent of MFX absorption remains unaffected by calcium-containing supplements, although the absorption rate may be slightly delayed [94,106,109].

**QT-Prolonging Agents:** An additive effect on QT interval prolongation may occur when MFX is co-administered with other agents known to affect cardiac repolarization. This interaction increases the risk of ventricular arrhythmias, including Torsade de Pointes. MFX should therefore be used with extreme caution in patients receiving any of the following [87,94,106]:

- **Class IA and III Antiarrhythmics:** e.g., amiodarone, sotalol, quinidine, and disopyramide [87,106].
- **Antipsychotics:** e.g., haloperidol, pimozide, and phenothiazines [87,106].
- **Tricyclic Antidepressants:** e.g., amitriptyline and imipramine [87,106].
- **Specific Antimicrobials and Antimalarials:** e.g., erythromycin, pentamidine, and halofantrine [87,106].
- **Other Agents:** e.g., cisapride, bepridil, and certain antihistamines (terfenadine and astemizole) [87,106].
- **Potassium-Depleting Agents:** Caution is also warranted in patients taking medications that induce hypokalemia, such as loop or thiazide diuretics, corticosteroids, laxatives, and amphotericin B, as electrolyte imbalances can further potentiate the risk of LZD- or FQ-induced cardiac arrhythmias [87,106].
- **Other drugs**: Co-administration of MFX with other FQs is contraindicated due to the heightened risk of cumulative toxicity. In the context of the BPaLM regimen, the integration of moxifloxacin requires careful consideration of overlapping toxicity profiles. Since bedaquiline and pretomanid are also associated with transaminase elevations, their co-administration may increase the risk of additive hepatotoxicity. Consequently, rigorous and serial monitoring of liver function tests (LFTs) is imperative to ensure patient safety and guide clinical management.

Potentiation of CNS adverse effects has been observed with concomitant NSAID use (e.g., ibuprofen and naproxen), necessitating clinical vigilance. Concurrent therapy with corticosteroids, such as prednisone, significantly elevates the risk of tendinopathy and tendon rupture; thus, such combinations should be avoided.

MFX may also augment the hypoprothrombinemic effect of warfarin, requiring rigorous monitoring of INR and prothrombin time. FQs increase serum cyclosporine levels and decrease serum mycophenolate concentration. When co-administered with multiple doses of rifampicin, MFX AUC decreases by approximately 30%. The clinical consequences of this are unknown, and no dose adjustment is recommended on co-administration. Additionally, MFX may alter the pharmacokinetics of theophylline, SSRIs, and duloxetine, potentially leading to theophylline toxicity, seizure activity, or serotonin syndrome. Dysglycemia—both hypo- and hyperglycemia—has been reported in patients on concomitant insulin or oral hypoglycemic agents, requiring frequent blood glucose monitoring [3,12,94,98,106,108,110].

### 3.6. MFX Specific Patients Populations

**Breastfeeding:** MFX is excreted into breast milk, so it presents a potential risk of neonatal exposure, including disruption of gut flora and musculoskeletal toxicity. Consequently, clinicians should prioritize alternative antibiotics with better-established safety profiles, particularly for non-life-threatening infections. However, according to the WHO, there are multiple case reports of FQ being used in humans safely during breastfeeding. The decision to use an FQ can be made only after consulting clinicians with considerable experience in managing tuberculosis [3,86,94,98].

**Pregnancy:** The use of **moxifloxacin (MFX)** during pregnancy requires a rigorous individualized risk–benefit analysis, as clinical data in pregnant women are limited. Animal models have demonstrated teratogenic potential, particularly during organogenesis in the first trimester. Reversible joint injuries have been reported in children receiving some quinolones; however, this effect has not been reported among exposed fetuses. The potential risk for humans is unknown. Therefore, its clinical application during pregnancy should be reserved for scenarios where therapeutic benefits outweigh the potential risks to fetal development. However, according to the WHO, there are multiple case reports of FQs being used in humans safely during pregnancy. The decision to use FQ can be made only after consulting clinicians with considerable experience in managing tuberculosis [3,86,94,98,106].

**Hepatic impairment:** FQs, including MFX, can be used without restrictions in patients with mild or moderate liver failure (Child–Pugh classes A and B). However, in cases of severe liver disease (Child–Pugh class C), as occurs with any other drug, patients should be closely monitored through clinical evaluation and laboratory testing. Routine monitoring of liver function tests, including serum transaminase—alanine transaminase (ALT) and aspartate transaminase (AST)—levels, is advised to detect any signs of adverse effects or changes in liver function during moxifloxacin treatment. Although hepatotoxicity associated with MFX is rare, individuals with pre-existing liver conditions may be at higher risk [3,94,98].

**Renal impairment:** Regarding its pharmacokinetic profile, moxifloxacin does not require dosage adjustments in patients with any degree of renal impairment, including advanced stages (CrCL < 30 mL/min) or those undergoing dialysis. Even though total renal clearance (including inactive metabolites) accounts for approximately 45% of the dose, these metabolites do not possess clinical relevance that would necessitate dose modification [3,94,98].

**Fertility:** No specific studies with MFX in humans have been conducted to evaluate effects on fertility; however, animal studies do not indicate impairment [100,106].

## 4. Bedaquiline (BDQ)

BDQ (Sirturo; Janssen Therapeutics, New Brunswick, NJ, USA), a diarylquinoline formerly known as R207910 and TMC207, was discovered by Koen Andries and his team in 2005 [105,106]. It was the first member of a novel drug class approved for tuberculosis (TB) treatment since rifampin in 1971 [111,112,113]. On 28 December 2012, the US Food and Drug Administration (FDA) granted accelerated approval for BDQ to treat DR-TB, following promising results from multiple preclinical and clinical trials [113,114,115,116]. Structurally, BDQ is the first in its class and presents a quilinolic central heterocyclic nucleus, with alcohol and amine side chains responsible for its antimycobacterial activity [111,112,113,117]. It is the only anti-TB drug that exhibits a potent inhibitory effect on several species of mycobacteria by targeting adenosine triphosphate (ATP) synthase (ATP synthase) [118,119]. BDQ exhibits bactericidal activity against both actively replicating and non-replicating mycobacteria. This broad spectrum of action is supported by its ability to eliminate bacilli across different microenvironments, a characteristic linked to its sustained affinity for its target under low-pH and low-proton motive force values. No cross-resistance has been found between BDQ and isoniazid, rifampin, ethambutol, pyrazinamide, streptomycin, amikacin, or moxifloxacin. There have been a few reports of cross-resistance with clofazimine [25,120].

In June 2013, the WHO issued its first interim guidance for BDQ use in adults with pulmonary MDR-TB, with a conditional recommendation and very little evidence. Due to limited safety data and observed excess mortality in early trials, the drug was recommended only when an effective regimen could not otherwise be designed. Its use was subject to five strict conditions: (a) treatment under closely monitored conditions; (b) proper patient inclusion (initially adults ≥ 18 only); (c) documented informed consent; (d) adherence to WHO-recommended MDR-TB regimen design principles; and (e) active pharmacovigilance and adverse event management. Bedaquiline is placed with anti-TB drugs belonging to Group 5, primarily because it does not belong to any of the other TB drug families and because, as of yet, there are limited data on its effectiveness and long-term safety in DR-TB treatment [121]. In 2014, the WHO maintained BDQ in Group 5 (anti-TB drugs with limited data on efficacy and/or long-term safety in the treatment of DR-TB) [122]. In May 2016, the WHO updated its MDR-TB management guidelines, reclassifying drugs into four categories (A, B, C, and D). Although BDQ was added to the WHO Model List of Essential Medicines, it was classified in Group D2 as a non-core agent, recommended only when conventional second-line drugs were insufficient or poorly tolerated [33]. A major policy shift occurred in August 2018, when the WHO reclassified BDQ as a Group A drug. This elevation established BDQ as a core component of long-course regimens for nearly all MDR-TB patients, facilitating the prioritized phasing-out of toxic injectable agents [34]. Following results from the TB-PRACTECAL and ZeNix trials, the WHO issued Rapid Communication in May 2022. The current standard of care for MDR/RR-TB is now a 6-month all-oral regimen consisting of BPaLM [13].

The recommended BDQ dosage for adults and adolescents at least 14 years old is as follows:

- Weeks 1–2: 400 mg (four tablets of 100 mg) once daily with food.
- Weeks 3–24: 200 mg (two tablets of 100 mg) three times per week with food (with at least 48 h between doses) for a total dose of 600 mg per week [3].

### 4.1. BDQ Mechanism of Action

Even though BDQ is closely related to FQs it displays no inhibitory effects on DNA gyrase and its mechanism of action differs [108]. Contrasting with other anti-TB drugs, BDQ aims to target the energy metabolism of mycobacterium by blocking the mycobacterial ATP synthase [123,124]. A ubiquitous enzyme located in the inner membrane of mycobacterial plasma mitochondria; the ATP synthase is an essential enzyme in synthesizing ATP and energy generation in all bacteria [125,126]. ATP production is necessary for the continued existence of all kinds of mycobacterium, either extracellular or intracellular, replicating or non-replicating, active or dormant and fermenting or non-fermenting [54]. Unlike many traditional anti-TB agents that require active cell division to be effective, BDQ’s bactericidal activity remains robust against persistent bacilli, a property that is fundamental to the success of the **BPaLM** regimen [126]. BDQ acts by inhibiting the function of ATP synthase by binding with a membrane-bound subunit of F1F0-ATP synthase, which inhibits the rotation and proton transfer of subunit-c encoded by the *atpE* gene, which is essential for enzyme action. This binding block proton pump activation and inhibits ATP synthase functioning. The blocking and binding of the c-subunit rotation results in ATP inhibition or the production of energy from the ATP synthase, which causes cell death [125,126,127]. In addition to the c-subunit, BDQ also aims to target the ε-subunit of F-ATP synthase by binding with Trp16 residue [128]. A key feature of BDQ is its high selectivity. It possesses a >20,000-fold higher affinity for mycobacterial ATP synthase compared to human mitochondrial ATP synthase. This extreme specificity minimizes target-based toxicity in human cells, as the binding pockets in human enzymes differ significantly from those in mycobacteria [123].

### 4.2. Mechanisms of Resistance

The primary mechanisms conferring resistance to BDQ include mutations in the *atpE* gene, which encodes ATP synthase; alterations in the *rv0678* gene, which regulates the MmpS5-MmpL5 efflux pump. Notably, mutations in this gene are associated with **cross-resistance between bedaquiline and clofazimine**, a factor that may compromise treatment outcomes in patients previously exposed to either drug. Additionally, mutations in the **pepQ** gene (*rv2535*) have been linked to low-level resistance to BDQ [57,117,121,122].

### 4.3. BDQ Pharmakokinetics

Bedaquiline (BDQ) exhibits a complex pharmacokinetic profile characterized by high lipophilicity and extensive tissue distribution. The drug is absorbed well following oral administration, reaching Cmax within 4–6 h (Tmax) independent of dose [114,117,129]. Nutritional status significantly impacts BDQ bioavailability; co-administration with a high-fat meal increases the mean area AUC by 2.0- to 2.4-fold compared to fasted states [117]. The drug is highly protein-bound (>99.9%) with a large apparent volume of distribution (approximately 164 L); however, it demonstrates poor penetration into the CNS [111,117]. At the recommended daily dose of 400 mg, BDQ reaches a Cmax of 5.5 mg/L and an AUC_0–24_ of 65 mg.h/L, with a systemic clearance (Cl) of approximately 6.2 L/h [130]. A defining feature of BDQ is its exceptionally long terminal half-life of approximately 5.5 months, driven by its slow redistribution from peripheral tissues back into the plasma [130]. While this persistence supports intermittent dosing (e.g., thrice weekly) after the initial loading phase, it also poses a risk: the “long tail” of sub-therapeutic concentrations may promote the emergence of resistance if the companion drugs in the regimen are discontinued prematurely [130,131]. In terms of microbiologic activity, the MIC for susceptible *M. tuberculosis* and MDR-TB strains typically ranges from 0.008 to 0.12 μg/mL [132]. This high potency, combined with its unique pharmacokinetics profile, underpins its efficacy in shortened regimens like BPaLM.

BDQ undergoes primary hepatic metabolism mediated by the cytochrome P450 3A4 (CYP3A4) isoenzyme, resulting in its *N*-monodesmethyl metabolite, M2. Although M2 retains antimycobacterial activity against *M. tuberculosis*, its potency is approximately five-fold lower than that of the parent compound [133,134]. In patients receiving therapeutic doses of BDQ, the plasma M2-to-BDQ ratio typically ranges from 0.25 to 0.30 [133,135]. Like the parent drug, M2 exhibits an exceptionally long terminal elimination half-life (t_1/2_), averaging 159 days (range: 69–407 days) following WHO-recommended dosing [133]. A second, quantitatively minor metabolite, M3 (N-didesmethyl), is formed via the N-demethylation of M2; however, M3 possesses negligible antimicrobial activity [133].

In vitro studies have indicated that both M2 and M3 exhibit greater cytotoxicity and phospholipidogenic potential than bedaquiline [117]. While cellular phospholipidosis has been associated with adverse effects such as QT interval prolongation, hepatotoxicity, and myopathy, in vivo clinical data suggest that the concentrations of M2 and BDQ achieved at Cmax do not typically reach the thresholds required to induce these toxicities [117]. Beyond the primary CYP3A4 pathway, isoforms such as CYP2C8 and CYP2C19 provide secondary metabolic routes, which may offer a buffering effect against drug–drug interactions (DDIs) involving CYP3A4 inhibition [117,136]. The pharmacokinetics of the BPaLM components are significantly influenced by individual patient factors. Bedaquiline and pretomanid are primary substrates of the cytochrome P450 3A4 (CYP3A4) isoenzyme; thus, pharmacogenetic variations in the *CYP3A* genes may lead to substantial inter-individual variability in drug exposure. Ethnicity and geographical region have also been identified as sources of variability in drug clearance and volume of distribution. Furthermore, sex-based differences play a clinical role, as female patients often exhibit higher plasma concentrations of bedaquiline, which may necessitate closer monitoring for QT interval prolongation [29,114,117,119,127].

BDQ elimination is predominantly fecal, with 75–85% of the dose recovered as the unchanged drug and 3.7–7.2% as the M2 metabolite within 24 h of administration. Clinical studies have demonstrated that renal excretion is negligible, with less than 0.001% of a 400 mg oral dose recovered unchanged in the urine [117,136]. Consequently, renal clearance plays a minimal role in the overall pharmacokinetic profile of the compound, suggesting that dose adjustments may not be required in patients with renal impairment.

### 4.4. BDQ Adverse Effects

BDQ’s safety profile is characterized by specific toxicities that necessitate rigorous clinical monitoring. According to the WHO Summary of Product Characteristics, the most frequent AEs, affecting more than 10% of patients, include QT prolongation (61%), nausea (54%), vomiting (54%), arthralgia (45%), elevated liver enzymes (30%), dizziness (18%), and headache (17%) [137].

**Cardiovascular Safety and QT Prolongation:** A primary clinical concern regarding BDQ is the dose-dependent **prolongation of the QT interval**, mediated by its inhibitory effect on the **hERG** (*human Ether-à-go-go-Related Gene*) potassium channels in cardiac myocytes [53,137,138,139]. This effect is largely attributed to the M2 metabolite, which, due to its exceptionally long half-life, can lead to delayed maximal QTc effects for up to 24 weeks after treatment initiation [53,137,138]. While the incidence of life-threatening arrhythmias such as *torsades de pointes* remains low, the risk increases significantly when BDQ is co-administered with other QTc-prolonging agents, such as clofazimine, delamanid, or FQs (e.g., LVX or MFX) [85,137,138]. Consequently, a baseline ECG is mandatory, followed by at least monthly assessments throughout treatment duration. Furthermore, serum electrolytes—specifically potassium, calcium, and magnesium—must be evaluated and corrected prior to and during therapy, as imbalances further predispose patients to cardiac events [137,139].

Adverse reactions to BDQ are listed below by system organ class (SOC) and frequency. Frequency categories are defined as follows: very common (at least 1 in 10), common (1 in 100 to 1 in 10) and uncommon (1 in 1000 to 1 in 100) [137].

- **Nervous system disorders**—very common: headache and dizziness [3,137].
- **Cardiac disorders**—very common: QT interval prolongation [3,137].
- **Gastrointestinal disorders**—very common: nausea and vomiting; common: diarrhea [3,137].
- **Hepatobiliary disorders**—very common: raised liver enzyme values [3,137].
- **Musculoskeletal and connective tissue disorders**—very common: arthralgia; common: myalgia [3,137].
- **Other side effects**—hemoptysis, chest pain, limb pain, joint pain, anorexia, rash, hyperuricemia, pancreatitis, and phospholipidosis [3,53,110].

### 4.5. BD Interactions

BDQ is metabolized in the liver by CYP3A4. Consequently, drugs that inhibit cytochrome CYP3A4 could result in increased concentrations of BDQ, which could increase toxicity, whereas drugs that induce CYP3A4 activity could result in reduced BDQ concentrations [117]. As a CYP3A4 substrate, and due to its high lipophilicity, BDQ causes moderate-to-high-risk drug–drug interactions with CYP3A4 inducers or inhibitors [80].

(a) **CYP3A4 inducers:** Co-administration of BDQ and moderate or strong CYP3A4 inducers, e.g., carbamazepine, efavirenz, etravirine, phenytoin, rifamycins (including rifampicin, rifapentine, and rifabutin), and St John’s wort (Hypericum perforatum), should be avoided. This is because CYP3A4 induction may reduce bedaquiline levels and therefore its therapeutic effect [117,137,140]. In an interaction study of single-dose BDQ and once-daily rifampicin (strong inducer) in healthy adults, bedaquiline exposure (area under the concentration–time plot, AUC) was reduced by around 50% [3,80,137].
(b) Inhibitors may not always necessitate dose adjustments, caution is required depending on the potency and duration of the interaction. Short-term co-administration of bedaquiline with potent CYP3A4 inhibitors, such as ketoconazole or clarithromycin, in healthy adults increased BDQ exposure (AUC) by approximately 22% and 14%, respectively [137]. However, a more pronounced and clinically significant increase in BDQ systemic exposure may occur during prolonged co-administration with potent CYP3A4 inhibitors, potentially increasing the risk of QT prolongation. Therefore, if long-term co-administration is required, frequent clinical monitoring and ECG assessments are recommended [137].
(c) **Other tuberculosis drugs:** Short-term co-administration of BDQ with isoniazid/pyrazinamide in healthy adults did not result in clinically relevant changes in the exposure (AUC) to BDQ, isoniazid or pyrazinamide. No dose adjustment of isoniazid or pyrazinamide is required during co-administration with BDQ. In a placebo-controlled clinical study involving patients with MDR-TB infection, the co-administration of BDQ had no major impact on the pharmacokinetics of cycloserine, ethambutol, kanamycin, ofloxacin, or pyrazinamide [112,123,130,137].
(d) **Antiretroviral medicines**: In an interaction study of single-dose BDQ and multiple-dose lopinavir/ritonavir in adults, exposure (AUC) to BDQ was increased by approximately 22%, while the AUC of lopinavir was unaffected [117,141]. Long-term co-administration of BDQ as part of a combination therapy and lopinavir/ritonavir in patients co-infected with HIV resulted in a mild increase in mean BDQ exposure at week 24 compared to a subgroup without HIV co-infection. Increases in plasma exposure to BDQ would be expected when co-administered with other ritonavir-boosted HIV protease inhibitors. There is currently no data to support a lowered bedaquiline dose during concomitant use with lopinavir/ritonavir or other ritonavir-boosted HIV protease inhibitors [137]. With the co-administration of BDQ with efavirenz, the AUC of BDQ decreased by 18%, while the Cmax was not affected [135]. As such, the use of efavirenz and protease inhibitors should be avoided in patients receiving BDQ [142,143]. Co-administration of bedaquiline and nevirapine in adults did not result in clinically relevant changes in BDQ exposure [123].
  The WHO recommends the use of two NRTIs with nevirapine or triple NRTIs to treat HIV in patients receiving BDQ-containing regimens. However, there are concerns that switching efavirenz-containing regimens to nevirapine-containing ones may reduce antiretroviral efficacy and increase the risk of viral failure and emergence of resistance [57,143,144]. Clinical data on the co-administration of BDQ and other antiretroviral agents in adults co-infected with HIV and DR-TB are not available [137].
(e) **QT interval-prolonging medicines**: BDQ is associated with a dose-dependent prolongation of the QT interval. When combined with other QT-prolonging agents, there is an additive or synergistic effect on cardiotoxicity. With concomitant use of BDQ and ketoconazole, a greater effect on QTc was observed [145]. Furthermore, QT elongation is exacerbated when bedaquiline is used in combination with other antibiotics, such as clofazimine, moxifloxacin, and macrolides. In addition, combining BDQ with clofazimine and FQs significantly increases the risk of the QTc interval exceeding **500 ms**, which is a biomarker for life-threatening arrhythmias like *torsades de pointes (Li)*. The use of BDQ in regimens containing any of these antimicrobial agents should be closely monitored [117,118,140,145]. However, in general, BDQ may have an additive or synergistic effect on QT prolongation when co-administered with other medicines that prolong the QT interval and frequent monitoring is recommended [3,137].

### 4.6. BDQ Specific Patient Populations

**Breastfeeding:** The safety of BDQ during breastfeeding has not been established in clinical trials, as lactating women were systematically excluded from early pivotal studies. Consequently, it is unknown whether BDQ or its metabolites are excreted in human milk. However, preclinical data from animal models have demonstrated that bedaquiline is present in the milk of lactating rats, reaching concentrations 6- to 12-fold higher than those observed in maternal plasma [133,137,146].

Given the drug’s high lipophilicity and exceptionally long half-life, there is a theoretical risk of significant drug accumulation in the breastfed infant [117]. Current WHO and FDA guidelines recommend that a decision should be made regarding whether to discontinue breastfeeding or to abstain from BDQ therapy, considering the importance of the drug to the mother and the potential benefits of breastfeeding to the infant [3,137,146].

**Pregnancy:** The use of BDQ during pregnancy presents a significant clinical challenge due to the historical exclusion of pregnant women from pivotal controlled trials [127,140]. Current WHO guidelines recommend that BDQ be used in pregnant women only when the potential benefits of treating MDR-TB outweigh the theoretical risks to the fetus [3,131]. Notably, preclinical reproductive toxicity studies in animal models (rats and rabbits) have shown no evidence of direct fetal harm, embryotoxicity, or teratogenicity, even at exposures significantly higher than those achieved with human therapeutic doses [3,137]. In such instances, a multidisciplinary approach is essential, incorporating close pharmacovigilance and long-term infant follow-up to monitor for potential delayed adverse effects, particularly regarding growth and neurodevelopmental milestones [3,137].

**Hepatic impairment:** BDQ should be used with caution because it is metabolized in the liver. No dosage adjustment is required in patients with mild-to-moderate hepatic impairment. It has not been studied in patients with severe hepatic impairment and should be used with extreme caution in such patients, and only when the benefits outweigh the risks. Clinical monitoring for BDQ-related adverse reactions is recommended [3].

**Renal impairment:** No BDQ dosage adjustment is required in patients with mild-to-moderate renal impairment. It should be used with caution in patients requiring peritoneal dialysis or hemodialysis. Therapeutic drug monitoring may be useful if available [3].

**Fertility:** No human data on the effect of BDQ on fertility are available. In female rats, there was no effect on mating or fertility with bedaquiline treatment; however, some effects occurred in male rats [137].

## 5. Pretomanid (PA)

Pretomanid (formerly PA-824), a nitroimidazopyran derivative, represents a milestone in pharmaceutical history as the first tuberculosis (TB) drug developed and registered by a non-profit organization, the **TB Alliance** (Global Alliance for TB Drug Development) [147,148,149]. The name “Pretomanid” honors Pretoria, South Africa, where much of its clinical development took place [150]. Following BDQ and delamanid, it became the third novel TB drug to receive approval from the US FDA and the European Medicines Agency (EMA) in over four decades [54,151].

The anti-TB effect of metronidazole was identified in the 1990s and is principally due to its activation under anaerobic conditions via nitro-reduction. This process generates reactive nitrogen and oxygen species that damage mycobacterial DNA and proteins [152]. The evolution of **pretomanid (PA-824)** originated from efforts to improve the limited tuberculosis activity of metronidazole and address the mutagenic profile of its potent precursor, **CGI 17341** [152,153]. Through a systematic evaluation of more than 300 structural analogs, PA was identified as a lead candidate, combining superior safety with robust antimycobacterial efficacy [152,153]. Pretomanid belongs to the nitroimidazole class, which also includes delamanid. While both drugs share similar mechanisms of action, pretomanid is the preferred component for the BPaLM regimen due to several clinical and pharmacological advantages. Unlike delamanid, pretomanid demonstrates a superior synergistic effect when combined with bedaquiline and linezolid, as evidenced in the landmark Nix-TB and TB-PRACTECAL trials. Furthermore, delamanid has been associated with a more pronounced risk of QT interval prolongation, which could potentially exacerbate the cardiotoxicity already associated with bedaquiline and moxifloxacin. These factors, combined with a simplified once-daily dosing schedule, establish pretomanid as the cornerstone for standardized short-course treatments [3,10,11,12,154,155,156,157,158,159,160,161,162].

PA features a unique bicyclic core—a nitroimidazole ring fused with an oxazine ring—that facilitates specific metabolic interactions with *MTB*. Its antimicrobial activity is driven by the nitro group (-NO_2_), which undergoes intracellular reduction to generate bactericidal reactive metabolites [154,155]. Pharmacologically, the trifluoromethoxy group (-OCF_3_) enhances lipophilicity, aiding cell wall penetration and in vivo absorption [154,155], while a hydrophobic side chain attached via an ether linkage optimizes solubility, bioavailability, and tissue distribution [154,155]. Collectively, these structural elements are essential to the drug’s efficacy and pharmacokinetic profile in treating MDR-TB [154,155].

On 14 August 2019, PA was approved by the US FDA in combination with BDQ and LZD for the treatment of adults with MDR-TB, or those resistant to isoniazid and rifampicin, who are treatment-intolerant or non-responsive to standard therapy; the recommended dose was 200 mg/day for 26 weeks [156]. It was subsequently approved by the European Medicines Agency (EMA; Amsterdam, The Netherlands), the Indian Health Authority (The Central Drugs Standard Control Organization [CDSCO]; New Delhi, India), and many other health authorities across the world [148,157]. In December 2022, the WHO updated the guidelines and recommended PA-based regimens for treating RR-/MDR-/pre-XDR-TB [13].

### 5.1. PA Mechanism of Action

PA is a prodrug that is metabolically activated by a nitroreductase enzyme, known as Ddn; it produces various active metabolites that are responsible for its other therapeutic actions, particularly nitric oxide induction. The nitroreductase enzyme (**Ddn**), which activates PA, is deazaflavin-dependent and relies on the reduced cofactor F_420_ (F_420_H_2_). This cofactor is regenerated via the F_420_-dependent glucose-6-phosphate dehydrogenase (Fgd1), a bacterial enzyme distinct from human G6PD [158,159,160]. The reduction in the nitro group on the imidazole ring leads to the formation of reactive des-nitro metabolites and the release of reactive nitrogen species [158,159,160]. **In aerobic conditions**, where bacteria are rapidly dividing, PA primarily acts as a cell wall inhibitor. It inhibits the biosynthesis of mycolic acids, specifically by blocking the oxidation of hydroxymycolates to ketomycolates. This essential lipid component depletion leads to defective cell wall formation and subsequent bacterial lysis. Recent studies also suggest that PA causes the toxic accumulation of methylglyoxal by targeting the pentose phosphate pathway, which further induces cellular arrest [80,161,162,163,164].

### 5.2. Mechanisms of Resistance

The genetic basis of PA resistance typically involves loss-of-function mutations in any of the six genes governing this activation system—ddn (rv3547): the gene encoding the primary nitroreductase; fgd1 (rv0407): the glucose-6-phosphate dehydrogenase responsible for recycling F420; and fbiA, fbiB, fbiC, and fbiD: a cluster of genes essential for the biosynthesis of the F420 cofactor [158,159,160,165].

Mutations in ddn are the most frequently reported in resistant isolates, followed by defects in the fbi biosynthetic pathway [166]. While these mutations often confer cross-resistance to delamanid due to the shared activation machinery, recent studies have identified specific polymorphisms, such as in the fbiD locus, that may differentially affect susceptibility levels to each drug [167]. Interestingly, while resistance is readily generated in vitro, its clinical prevalence remains low, potentially due to the fitness cost associated with F420 cofactor loss, which plays a protective role against oxidative stress in the bacteria [168].

### 5.3. PA Pharmacokinetics

Following a single oral administration of 200 mg, PA exhibits significant exposure variability depending on the prandial state. In healthy volunteers under fasted conditions, the mean Cmax is approximately 1.1 µg/mL, with an AUC∞ of 26.3 μg·h/mL [163,164]. Bioavailability is markedly enhanced when PA is administered with a high-fat, high-calorie meal, which increases the Cmax to 2.0 µg/mL (an 80% increase) and the AUC∞ to 44.9 μg·h/mL (an 88% increase) [169,170]. The median time to reach peak concentration (Tmax) typically ranges between 4 and 5 h, regardless of food intake. However, absorption is more consistent in the fed state, underscoring the clinical necessity of administering PA with food to reach the pharmacodynamic targets required for treating MDR-TB [164,165]. PA is approximately 86% protein-bound, primarily to albumin, and exhibits an apparent volume of distribution (Vd/F) between 92 and 180 L [148,163]. Due to its lipophilic nature, the drug demonstrates good brain tissue penetration in preclinical and early human imaging studies; however, CSF levels are typically lower, and its clinical efficacy in tuberculous meningitis requires further validation [154,170]. The terminal elimination half-life (t1/2) remains relatively constant at approximately 16 to 20 h, a profile that supports a once-daily dosing frequency [154,170].

PA undergoes extensive hepatic metabolism through multiple pathways, involving both oxidative and reductive processes. Unlike many other anti-tuberculosis agents, it is not significantly metabolized by CYP3A4. Instead, the primary metabolic route involves the reduction in the nitro group, a process whose activation is mediated by the deazaflavin-dependent nitroreductase (Ddn) in *MTB*, and by various human reductases for its systemic clearance [154,159]. In vitro studies using human liver microsomes and hepatocytes indicate that approximately 20% of PA metabolism is attributed to CYP3A4, while other pathways, including non-enzymatic reduction, account for the remainder [147,171,172]. Following oral administration, PA excretion is primarily shared between the renal and fecal routes. Approximately 53% of a radiolabeled dose is recovered in the urine and 38% in the feces, mainly as metabolites; less than 1% of the dose is excreted unchanged in the urine [169,171,173]. Given the minimal renal clearance of the unchanged parent drug, dose adjustments are generally not required for patients with mild-to-moderate renal impairment [163,165,167,169,171,173]. PA demonstrates potent in vitro activity against both drug-susceptible and -resistant strains of the *Mycobacterium tuberculosis* complex (MTBC). For the majority of susceptible (wild-type) and MDR isolates, the MIC typically ranges from 0.015 to 0.25 µg/mL [167,174]. Recent large-scale surveillance studies have confirmed that PA maintains its efficacy regardless of resistance to first-line drugs, such as isoniazid and rifampicin, confirming a lack of cross-resistance [151,154].

### 5.4. PA Adverse Effects

PA is generally well-tolerated, with short-term monotherapy (50–1200 mg) showing no serious drug-attributable adverse effects [35,175]. While isolating the individual toxicity of PA within combination regimens remains challenging, the most frequent adverse events reported in clinical trials include gastrointestinal symptoms (28.4%), hepatic disorders (25.5%), elevated transaminases (19.2%), dermatologic disorders (16.6%), and headache (11%) [176]. Notably, only gastrointestinal symptoms—predominantly mild-to-moderate nausea, vomiting, and dyspepsia—exhibit a direct correlation with higher systemic drug exposure [154,176]. Hepatotoxicity specifically attributable to PA is low (2.2%), though rates increase to 5.6–11.7% in combination regimens, suggesting the exacerbating effect of companion drugs [171,177]. Additionally, PA causes a dose-dependent increase in serum creatinine. This phenomenon results from the inhibition of tubular secretion rather than a reduction in the glomerular filtration rate (GFR) and is fully reversible upon treatment cessation [170,178]. Regarding cardiovascular safety, although early analyses noted a minor correlation between plasma levels and QTc intervals, subsequent studies have found not clinically meaningful QTc prolongation attributable to the drug [175]. In preclinical animal models, concerns were raised regarding ocular disorders and male reproductive toxicity. However, a comprehensive review of four clinical trials reported no significant changes in male hormones, suggesting no association between PA and testicular toxicity in humans [3,179,180]. Further data from reproductive safety studies focused on sperm counts confirm that PA does not negatively impact human male reproductive function [3,175,181]. Similarly, while monkey models initially suggested a risk of delayed cataract formation, prospective ocular monitoring in Phase 2 and 3 trials (including NiX-TB) has consistently failed to demonstrate drug-induced lens opacities in human subjects [3,11,182].

### 5.5. PA Interaction

PA exhibits a distinct pharmacokinetic profile with specific interaction potentials involving CYP3A4 and membrane transporters.

**Effects of Co-administered Drugs on PA:** As PA is partially metabolized by CYP3A4, its systemic exposure is significantly influenced by CYP3A4 inducers. Co-administration with rifampicin and efavirenz has been shown to reduce Pretomanid AUC_0–24 h_ by 66% and 35%, respectively [151,164,177]. Consequently, the concurrent use of moderate-to-strong CYP3A4 inducers—including carbamazepine, phenytoin, rifamycins, and St. John’s wort—should be avoided to prevent sub-therapeutic drug levels and potential treatment failure [151,165,177]. Notably, co-administration with ritonavir-boosted lopinavir resulted in a 17% reduction in PA AUC_0–24 h,_ which is generally not considered clinically significant [157,183].

**Effects of PA on Other Medications:** PA may modulate the pharmacokinetics of various substrates through enzyme induction and transporter inhibition:

- **CYP Isoenzymes:** In vitro data suggest that PA induces CYP2C8, with inconclusive evidence regarding CYP2C9 and CYP2C19 [157,183]. Clinicians should monitor for reduced efficacy of substrates such as warfarin, paclitaxel, and mephenytoin when co-administered with PA.
- **Transporters:** Pretomanid inhibits the organic anion transporter 3 (**OAT3**) in vitro, potentially increasing the plasma concentrations of substrates like ciprofloxacin, methotrexate, and indomethacin [157,183]. Furthermore, inhibition of BCRP, OATP1B3, and P-gp may increase exposure to sensitive substrates, including statins, digoxin, and dabigatran [157,183]. Close monitoring for adverse reactions related to these co-administered agents is recommended, and dosage adjustments may be required.

### 5.6. PA Specific Patients Populations

**Breastfeeding:** There is currently no clinical data regarding the excretion of PA or its metabolites in human milk, effects on breastfed infants, or the impact on milk production. Consequently, the safety of PA during breastfeeding remains unestablished [171,183]. Due to the lack of human safety data and the potential for serious adverse reactions in nursing infants—including hepatotoxicity and potential developmental risks—the WHO and the FDA recommend that a decision be made whether to discontinue breastfeeding or to abstain from PA therapy [3,171,183].

**Pregnancy:** The safety of PA during pregnancy remains unestablished [3,157,171,183]. In alignment with the WHO 2025 recommendations [3], the BPaLM regimen is not recommended for pregnant or breastfeeding women. This exclusion is primarily due to the limited safety and pharmacokinetic data for PA in these special populations [3].

**Hepatic impairment:** The effect of hepatic impairment on the safety, effectiveness, and pharmacokinetics of PA is not known [3,171,183]. As PA-based regimens (e.g., BPaLM) can induce treatment-emergent transaminase elevations, baseline and monthly hepatic function monitoring should be a requirement for clinical management [154,170].

**Renal impairment:** The effect of renal impairment on the safety, effectiveness, and pharmacokinetics of PA is not known [3,164,177]. Clinical studies demonstrate that Cmax and AUC are not significantly altered by mild, moderate, or severe renal impairment; therefore, no dosage adjustment is required for these populations [172,178,179,180,181,182,183,184].

## 6. Conclusions

The BPaLM regimen represents a significant advancement in the therapeutic management of RR/MDR-TB. By combining four agents with complementary mechanisms, including the inhibition of mycobacterial ATP synthase (bedaquiline), cell wall biosynthesis and respiratory metabolism (pretomanid), protein synthesis (linezolid), and DNA-gyrase-mediated replication (moxifloxacin), this approach demonstrates high treatment success rates in eligible patients.

However, the clinical implementation of this regimen is not universal and remains contingent upon rigorous pharmacological oversight. Its use is limited to cases without documented fluoroquinolone resistance, and clinicians must actively manage dose-dependent toxicities. Specifically, linezolid-associated neuropathy and myelosuppression often necessitate dose reductions, while bedaquiline- and moxifloxacin-induced QTc prolongation require persistent monitoring. Furthermore, the hepatic safety profile of pretomanid, particularly in patients with pre-existing impairment, remains to be fully established. The clinical management of this combination requires a strategic approach to these overlapping toxicities. Effective strategies include rigorous electrolyte supplementation (K^+^, Mg^2+^) and a standardized dose-reduction protocol for linezolid to mitigate cumulative neurotoxicity without compromising efficacy.

Following WHO 2025 updates, the use of BPaLM in pregnant and breastfeeding populations remains restricted. Due to limited pretomanid safety data, these patients should be treated with alternative validated regimens after a rigorous risk–benefit assessment. From a public health perspective, the successful global scale-up of BPaLM hinges on the integration of active drug safety monitoring and management (aDSM) frameworks. In high-burden, resource-limited settings, the transition to this shortened regimen must be supported by robust pharmacovigilance systems to detect rare but serious adverse events. Furthermore, ensuring equitable access to bedaquiline and pretomanid, alongside the implementation of rapid molecular drug-susceptibility testing, is vital to prevent the emergence of further resistance and to ensure that the benefits of this therapeutic breakthrough reach the most vulnerable populations.

Finally, future research must transition from controlled trials to real-world adherence studies to evaluate treatment feasibility and patient retention. There is an urgent need for long-term outcome data (beyond the standard 6-month follow-up) to monitor for late relapses and the risk of resistance during the bedaquiline ‘long tail’. Optimizing dosing and safety protocols for special populations, particularly children and pregnant women, through targeted pharmacokinetic (PK) modelling and dedicated clinical cohorts, remains a priority to ensure the global sustainability and reach of this shortened therapeutic strategy.

## Figures and Tables

**Table 1:** Pharmacological and clinical profile of the BPaLM regimen components.

Component	Brief History	Mechanism of Action	Main Adverse Effects	Key Drug Interactions	Monitoring for AEs	Managing Adverse Reactions
Bedaquiline	First-in-class diarylquinoline approved by the FDA in 2012	Inhibits mycobacterial ATP synthase, disrupting energy production	Increase risk of QTc prolongation: may increase the risk of ventricular arrhythmias, hepatotoxicity: risk of liver enzyme elevation, chest pain	Strong cytochrome P450 3A4 (CYP3A4) inducers (e.g., Rifampicin) markedly reduce exposure	Baseline and monthly ECG; monthly LFTs (ALT/AST, bilirubin)	Avoid administration with other strong QT prolongers drugs. Discontinue if QTc > 500 ms or transaminases > 5× ULN (upper limit of normal).
Pretomanid	Developed by TB Alliance; US Food and Drug Administration (FDA)/European Medicine Agency (EMA) approved (2019–2020)	Prodrug; inhibits mycolic acid (aerobic) and induces respiratory poisoning (anaerobic)	Gastrointestinal (GI) symptoms, hepatotoxicity: potential for liver injury, reversible creatinine increase	Avoid strong CYP3A4 inducers; potential interactions with OAT3 (organic anion transporter 3) substrates	Monthly LFTs; baseline and monthly Serum Creatinine; inform patients about signs of hepatic dysfunction (jaundice, dark urine)	Administer with food to avoid GI symptoms and ensure adequate absorption. Creatinine elevation usually requires no action.
Linezolid	Repurposed oxazolidinone; dose-optimized in ZeNix trial	Inhibits protein synthesis by binding to the 23S rRNA (50S subunit)	Peripheral and optic neuropathy, myelosuppression (anemia, thrombocytopenia), lactic acidosis	Serotonergic agents (risk of Serotonin Syndrome); Monoamine oxidase inhibitors	Monthly complete blood Count (CBC); clinical screening for numbness; visual acuity tests; regular neurological exams for signal of paresthesia	Dose reduction (e.g., 600 mg to 300 mg) or interruption. Pyridoxine (B6) prophylaxis.
Moxifloxacin	4th-gen fluoroquinolone; added to BPaL to form BPaLM	Inhibits DNA gyrase and topoisomerase II, preventing DNA replication	QTc prolongation, risk of tendinitis/tendon rupture, peripheral neuropathy, and CNS effects (seizures, hallucinations); may exacerbate myasthenia gravis; dysglycemia	Cations (antacids/Fe/Ca) reduce absorption; other QT-prolonging drugs	Baseline and monthly ECG; assessment for joint/tendon pain	Discontinue if tendon inflammation occur, or neurological symptoms. Monitor electrolytes (K^+^, Mg^2+^). Avoid in patients with a history of myasthenia gravis or known CNS disorders.

## Data Availability

No new data were created or analyzed in this study. Data sharing is not applicable to this article.
